# Low-Cost Advanced Hydrogels of Calcium Alginate/Carbon Nanofibers with Enhanced Water Diffusion and Compression Properties

**DOI:** 10.3390/polym10040405

**Published:** 2018-04-04

**Authors:** Mar Llorens-Gámez, Ángel Serrano-Aroca

**Affiliations:** 1 Escuela Técnica Superior de Arquitectura, Universitat Politècnica de València, Camí de Vera s/n, 46022 Valencia, Spain; malloga1@arq.upv.es; 2 Facultad de Veterinaria y Ciencias Experimentales, Universidad Católica de Valencia San Vicente Mártir, C/Guillem de Castro 94, 46001 Valencia, Spain

**Keywords:** carbon nanofibers, calcium alginate, composite hydrogels, water diffusion, compression performance, Raman spectroscopy, electron microscopy

## Abstract

A series of alginate films was synthesised with several calcium chloride cross-linker contents (from 3 to 18% *w/w*) with and without a very low amount (0.1% *w/w*) of carbon nanofibers (CNFs) in order to reduce the production costs as much as possible. The results of this study showed a very significant enhancement of liquid water diffusion and mechanical compressive modulus for high calcium chloride contents when this minuscule amount of CNFs is incorporated into calcium alginate hydrogels. These excellent results are attributed to a double cross-linking process, in which calcium cations are capable of cross-linking both alginate chains and CNFs creating a reinforced structure exhibiting ultrafast water diffusion through carbon nanochannels. Thus, these excellent results render these new alginate composites very promising for many bioengineering fields in need of low-cost advanced hydrogels with superior water diffusion and compression properties.

## 1. Introduction

Hydrogels are currently employed in a broad range of industrial applications due to their excellent hydrophilic properties [1]. Alginate hydrogels are formed when divalent cations of calcium bridge sodium alginate (SA) polymer chains composed of (1–4)-linked β-d-mannuronic acid (M) and α-l-guluronic acid (G) blocks in different proportions and sequences depending on the source of alginate [2,3]. This cross-linking of the calcium cations occurs through the G blocks arranged in a block-wise fashion, thus forming the well-known egg-box structure [4]. Alginates have already been approved by the US Food and Drug Administration (FDA) for human use as wound dressing material in biomedical applications [5] due to their non-toxicity, biodegradability, biocompatibility and relative economic cost in comparison with other biopolymers. Therefore, these hydrogels have a wide range of applications in diverse fields, such as water decontamination [6], plastic packaging [7] and bioengineering [8,9]. However, calcium alginate, like most hydrogels, is very brittle [10], and is thus in need of reinforcing strategies to increase exponentially their potential applications. Thus, several methods to enhance the mechanical properties of hydrogels have been developed so far: reinforcement through copolymerisation with hydrophobic monomers [11], interpenetrating polymer networks (IPNs) [12], rise of cross-linking degree [13,14], double cross-linked networks [15], incorporation of nanofibre mats [16], self-sorting [17], plasma-induced polymerisation onto a hydrophobic porous polymer [18,19,20]. More recently, seemingly even more successful methods include the reinforcement of hydrogels by incorporation of carbon materials such as graphene oxide (GO) [21,22] and carbon nanofibers (CNFs) [23,24]. However, compared with GO, CNFs have much lower cost and much more electrical conductivity, which can be exploited to fabricate conductive composite materials [25,26]. Carbon nanofibers consist of curved graphene layers in the form of quasi one-dimensional (1D) filaments [27], promising to revolutionise several fields in material science [28] due to their excellent chemical, mechanical and electrical properties [29,30,31]. Thus, these curved graphitic materials are nowadays employed in industrial fields such as polymer additives, gas storage materials, and catalyst supports [32]. Additionally, it has been demonstrated that composites with CNFs are able to promote cardiomyocyte growth [33] and neural regeneration [34] in biomedicine.

Another conductive carbon material, chemically very similar to CNFs, carbon nanotubes (CNTs) have also been used to reinforce hydrogels [35,36,37]. However, CNFs have lower cost and higher purity level than CNTs, which can result in easier processing of nanocomposites [38].

On the other hand, enhancement of water diffusion in hydrogels is also very desirable in regenerative medicine because it increases cell survival [39] and in bioprocess industries, in which their production depends on the mass transport to the polymer biocatalysts with immobilised cells or enzymes [9]. Since it has been reported that liquid water penetrates graphene-based nanochannels with a diffusion coefficient 4–5 faster than in the bulk case [40], and water sorption studies in carbon membranes composed of aligned carbon nanotubes [41,42] or GO [43,44] have shown ultrafast diffusion of water through their hydrophobic graphitic nanochannels serving as pores, we assume that a significant enhancement of water diffusion in calcium alginate could be achieved with the incorporation of carbon nanofibers due to its similar chemical structure. However, only a very small amount (0.1% *w/w*) of CNFs was utilised in the synthesis in order to reduce production costs as much as possible and we hypothesize that this low nanofilling can enhance water diffusion and compression performance of alginate hydrogels, depending on the amount of Ca^2+^ present in the reactive mixture. Furthermore, diverse divalent cations have been shown to be capable of interconnecting or cross-linking nanomaterials such as GO nanosheets [45,46,47] or CNTs [48], and even chemically modified graphene (CMG) with CNTs [49] through their oxygen functional groups located at the edges and on the basal planes.

Therefore, we assume that 3D networks of CNFs can be cross-linked by metal ion coordination chemistry, as has been reported for the other nanomaterials, inside the alginate matrix when the amount of calcium atoms is high. We believe that when using high calcium chloride contents, the liquid water diffusion and compression properties of calcium alginate hydrogel films must be enhanced because of this double cross-linking process, which can achieve significant reinforcement due to the high degree of connection between the carbon nanofibers inside the cross-linked alginate polymer matrix with carbon nanochannels available for ultrafast water diffusion.

## 2. Materials and Methods

### 2.1. Materials

The sodium alginate (Panreac AppliChem, Darmstadt, Germany) was analysed by ^1^H-NMR and Gel Permeation Chromatography (GPC). The results of this characterisation showed an average molar mass of 142.000 g/mol and a mannuronate/guluronate ratio of 1.56. Calcium chloride (≥93.0%, Sigma-Aldrich, Saint Louis, MO, USA) and CNFs (Graphenano, Yecla, Spain) were utilised as received.

### 2.2. Synthesis

A series of alginates with several calcium chloride cross-linker contents (3, 6, 12 or 18% *w/w* with respect to the mass of SA) and 0.1% *w/w* of CNFs were synthesised using a procedure based on the direct mixing method [50]. Thus, 0.25 grams of SA were dissolved into 22 mL of an aqueous dispersion with 0.001% *w/v* of CNFs and was magnetically stirred for 1 h at 24 ± 0.5 °C. The required amount of calcium chloride was separately dissolved in 10 mL of distilled water and then mixed thoroughly with the former CNFs/SA mixture under magnetic stirring. Subsequently, the final solution was cast onto a Petri dish and left in an oven at 37 °C for 24 h to form a thin film. Finally, the film was peeled off from the mould and vacuum dry at 60 °C.

Another series of alginates with the same calcium chloride cross-linker contents (3, 6, 12, and 18% *w/w*) without CNFs, hereafter referred to as A3, A6, A12 and A18, was prepared following the same procedure, but directly solving only 0.25 grams of SA in 22 mL of distilled water. The composite hydrogels will be named hereafter by adding -CNFs to the corresponding alginate sample name (A3, A6, A12 or A18).

### 2.3. Characterisation

#### 2.3.1. Water Sorption and Diffusion

Water immersion experiments were performed in triplicate to study, in a reproducible manner, water sorption and diffusion at 24 ± 0.5 °C. Thus, the samples were vacuum-dried at 60 °C and then immersed in liquid water to be weighted at selected time intervals after drying with filter paper the water drops present on their surfaces.

#### 2.3.2. Electron Microscopy

A FEI Tecnai G^2^ F20 (Hillsboro, OR, USA) 200 kV high-resolution transmission electron microscope (HR-TEM) was employed to observe the CNFs dispersed in ethanol placing one drop on a TEM grid with 300 mesh coated in carbon film for five minutes. This electron microscope is equipped with energy-disperse X-ray spectroscopy (EDS) and was used for the elemental analysis of the CNFs at 20 kV. The alginate composite hydrogels with 0.1% *w/w* of CNFs were observed in a JEM-1010 (JEOL, Tokyo, Japan) 100 kV transmission electron microscope (TEM). Ultrathin sections (60 nm) were prepared using a Leica Ultracut UC6 ultramicrotome (Leica Mikrosysteme GmbH, Wien, Austria) and a Diatome diamond knife (Diatome Ltd., Bienne, Switzerland). The specimens were placed on TEM grids with 300 mesh coated in carbon film.

#### 2.3.3. Raman Spectroscopy

A Renishaw inVia (Wotton-under-Edge, UK) confocal micro-Raman spectrometer with an argon ion laser at 633 nm was utilised to perform Raman scans from 1000 to 3000 cm^−1^ with ×20 lens at 600 L·mm^−1^ grating. Sample preparation consisted of depositing the samples onto a glass substrate.

#### 2.3.4. Compression Testing

A texture analyser (Stable Micro System, TA-XT plus, Surrey, UK) with a 50 N load cell was used to conduct compression testing at 0.06 mm·s^−1^ from 0% to 100% strain at 24 ± 0.5 °C. Cylindrical samples 10 mm in diameter were vacuum-dried at 60 °C for 24 h before starting the tests. Six specimens were tested for each kind of sample, and their thickness and diameter were measured with a digital electronic calliper (ACHA, Éibar, Spain).

## 3. Results and Discussion

### 3.1. Water Sorption and Diffusion

**Figure 1:**
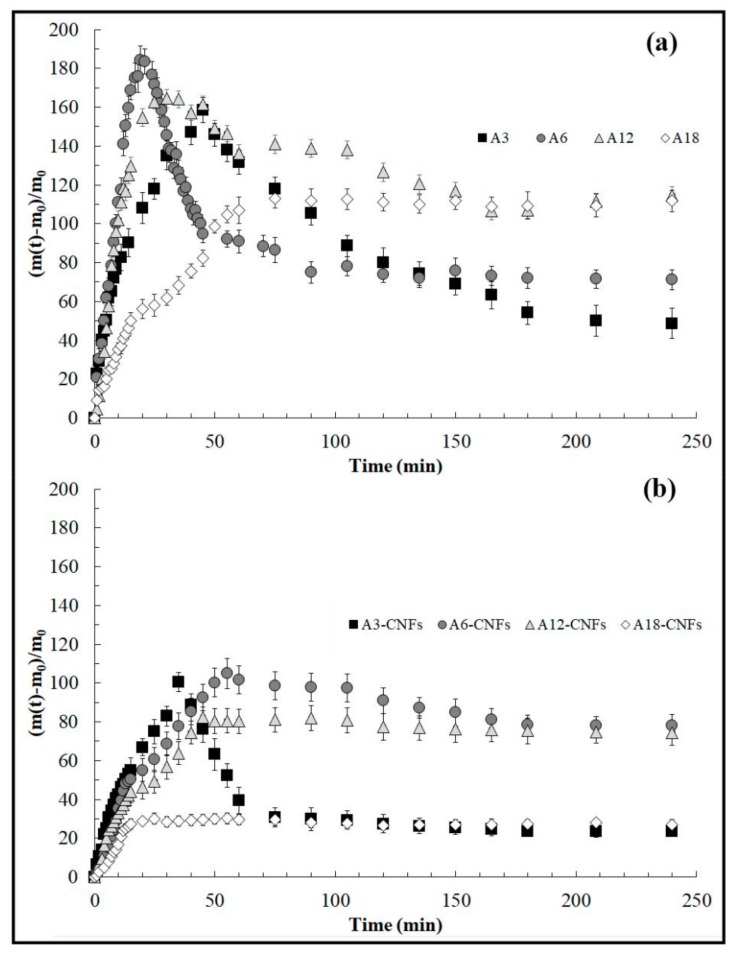
Water contents (grams of absorbed water per grams of dry sample) at 24 ± 0.5 °C represented as mean ± standard deviation for alginate hydrogels synthesised with several calcium chloride contents (**a**) and with also the addition of 0.1% *w/w* of CNFs (**b**).

Water sorption experiments exhibited a significant reduction of water sorption in the calcium alginate hydrogels with increasing cross-linker content as expected and surprisingly also by the small addition of 0.1% *w/w* of CNFs (see Figure 1).

The alginate hydrogels with lower cross-linker content, A3 and A6, shows the highest differences between the maximum and the last water uptake (see Figure 1a) due to the fact that they have more uncross-linked alginate chains available for dissolution during the water immersion experiment. The same phenomenon occurs with the A3-CNFs composite sample (see Figure 1b). However, this effect is attenuated in the A6-CNFs composite hydrogel, indicating some kind of additional cross-linking. In good agreement with these results, the sample A12-CNFs, which possess higher cross-linker content, exhibits almost no difference between the maximum and the last water uptake in comparison to A12. Furthermore, the pronounced reduction of equilibrium water sorption observed in A18-CNFs in comparison with A18 is remarkable, and reinforces the hypothesis of much higher cross-linking occurring in these composite samples with 0.1% *w/w* of CNFs.

According to the Fick’s law, water diffusion in hydrogels can be analysed with Equation (1).

(1) $$\frac{\Delta m_{1,t}^{}}{\Delta m_{1,\infty }^{}}\approx 4{\left(\frac{Dt}{\pi \cdot l_{}^{2}}\right)}^{\frac{1}{2}}$$

where Δ*m*_1*,t*_ and Δ*m*_1*,∞*_ are the weight increments at time *t* and at equilibrium, *l* is the sample thickness and *D* the diffusion coefficient. The maximum water contents of Figure 1 were taken as equilibrium values to calculate Δ*m*_1*,∞*_. The representation of all the Δ*m*_1*,t*_/Δ*m*_1*,∞*_ vs. *t*^1*/*2^*/l* plots showed a non-Fickian sorption mechanism [51] in these alginate hydrogels with and without CNFs, which is in good agreement with earlier studies carried out with liquid and vapour water in alginate-based hydrogels [52,53,54,55] and in alginate composites hydrogels with other carbon materials [21,56]. However, an apparent diffusion coefficient can be determining by linear regression fitting of Equation (1) for low values of time *t* or Δ*m*_1*,t*_/Δ*m*_1*,∞*_ < 0.5 to compare liquid water diffusion behaviour in all these similar polymeric systems.

**Figure 2:**
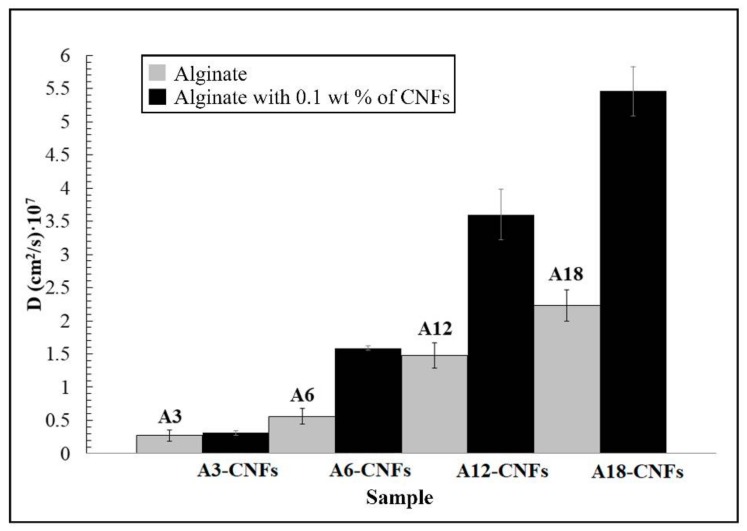
Apparent diffusion coefficients (*D*) of liquid water in the alginate hydrogels (grey columns) and in the composite alginate hydrogels with 0.1% *w/w* of CNFs (black columns) with the indicated cross-linker contents (3, 6, 12 and 18% *w/w*). Data are represented as mean ± standard deviation.

The results of this study showed a significant enhancement of water diffusion in calcium alginate composite hydrogels with 0.1% *w/w* of CNFs as compared with the corresponding alginate hydrogels with a similar cross-linker content (see Figure 2). However, this enhancement of water diffusion is attenuated for the lowest cross-linker content. The increase of cross-linker content also produced a significant increase of the water diffusion coefficient probably due to fact that cross-linking between l-guluronic acid (G) blocks must form some kind of discontinuities in the dry state through which water can penetrate faster.

### 3.2. Electron Microscopy and Raman Spectroscopy

**Figure 3:**
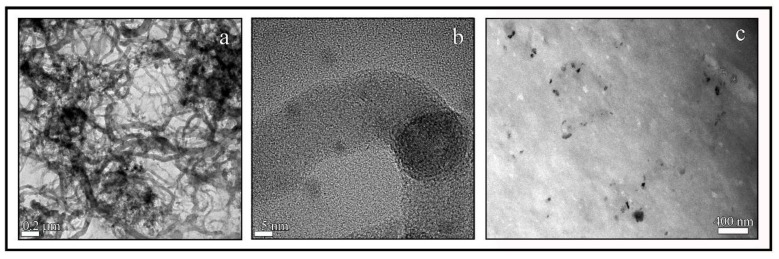
HR-TEM of the CNFs at two magnifications (**a**,**b**) and TEM of A18-CNFs (**c**).

The CNFs employed in this study exhibit a morphology of carbon nanofibres with irregular diameters of approximately 10–150 nm and lengths varying from a few nm to some µm (see Figure 3a,b).

**Figure 4:**
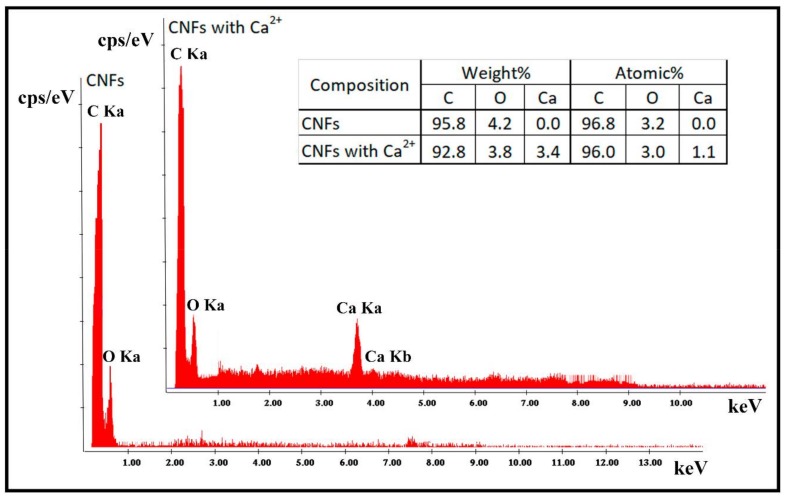
Elementary composition of the carbon nanofibers with and without cross-linking with Ca^2+^ measured by EDS at 20 kV.

It is of note that some black spots are present in the HR-TEM micrograph (Figure 3a). Thus, in order to confirm that these spots are only carbon atoms, the atom-atom length was measured in these black points, and the results showed an atom-atom distance of 3.42 Å, which corresponds to that of the van der Waals radius of carbon [57]. Furthermore, the EDS analysis supported these results because it showed that the elementary composition of CNFs is composed mostly of carbon atoms and a low % *w/w* of oxygen atoms (Figure 4). Figure 3c shows the morphology of the A18-CNFs composite hydrogel composed of alginate (light phase) and carbon nanofibers (dark phase) randomly distributed. It is remarkable that only the large CNFs or cross-linked CNF networks can be appreciated by electron microscopy because they are embedded in the alginate polymer matrix. The TEM morphology of this sample is not very different from those of the other composite hydrogels synthesised with less cross-linker content (images not shown). These connected structures of carbon nanofibers do not allow the A18-CNFs sample to close all of its porosity in the drying process, and some nanochannels are formed in some parts of the sample (see the dark phase of Figure 3c), similar to that which we have recently reported occurs with cross-linked graphene oxide nanosheets in alginate [21]. Thus, water can penetrate very fast through carbon nanochannels [58], increasing the water diffusion coefficient (Figure 2). However, it is much more difficult for water to diffuse in sample A18, because it is non-porous in the dry state.

**Figure 5:**
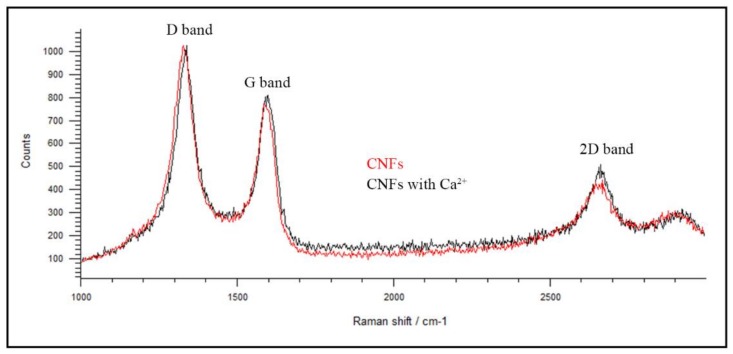
Raman spectra of CNFs and CNFs cross-linked with calcium cations.

In order to ensure that there is a chemical coordination reaction between divalent calcium cations and carbon nanofibers, EDS analysis (Figure 4) and Raman spectroscopy (Figure 5) was performed after dispersing CNF powder in distilled water and subsequent mixing with a 10% *w/v* calcium chloride solution with continuous magnetic stirring at 37 °C following the same procedure described in [45] for graphene oxide nanosheets. Thus, Figure 4 shows that the carbon nanofibers after cross-linking with calcium chloride present an elementary composition with 3.4 weight % or 1.1 atomic % of calcium atoms, which is in good agreement with our hypothesis that 3D networks of CNFs can be cross-linked by metal ion coordination chemistry, as has been reported for other nanomaterials also possessing oxygen-containing functional groups [45,46,47,48,49].

Raman spectroscopy is commonly utilised to obtain structural information about defects and ordered/disordered of carbon nanomaterials. The Raman spectrum of CNFs exhibits a strong D band at ~1350 cm^−1^ and a broad G band at ~1580 cm^−1^ [59]. The D band is attributed to a disordered band due to structural defects, edge effects and dangling sp^2^ carbon bonds breaking symmetry. The defect/disordered carbon structure is usually measured with the D band/G band intensity ratio (*I*_D_/*I*_G_) [60,61]. Thus, Figure 5 shows that after the cross-linking reaction of CNFs with divalent cations of Ca^2+^, the *I*_D_/*I*_G_ ratio decreases from 1471 to 1271 confirming a significant increase of order of the carbon structures due the cross-linking obtained with the calcium cations by coordination chemistry. In addition, the increase of intensity of the 2D band at ~2690 cm^−1^, supports these results, demonstrating higher degrees of order [59] achieved after the coordination reaction.

### 3.3. Compression Performance

**Figure 6:**
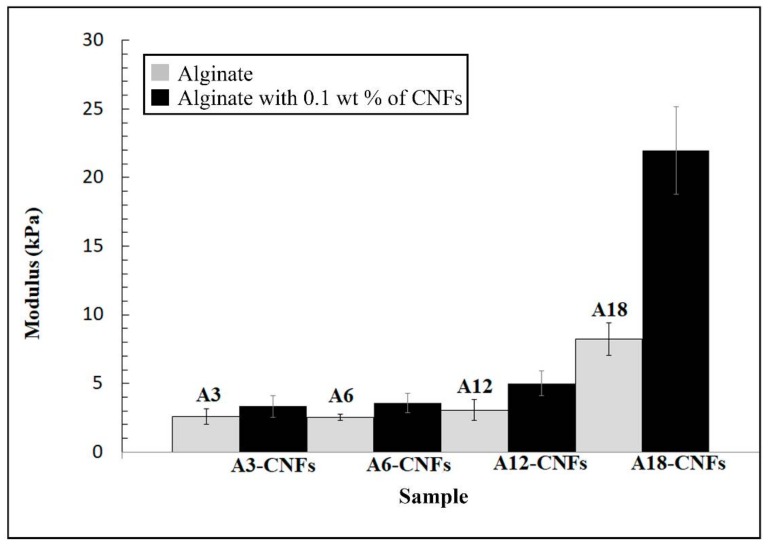
Mechanical compressive modulus (mean ± standard deviation) of alginate hydrogels (grey columns) and alginate composite hydrogels with 0.1% *w/w* of CNFs (black columns) cross-linked with increasing amounts of calcium chloride (3, 6, 12 and 18% *w/w*).

The effect of the addition of 0.1% *w/w* of CNFs into the alginate polymer hydrogels became more and more pronounced with increased weight percentage of calcium chloride (see Figure 6). Hence, the composite sample A18-CNFs exhibited a compressive modulus almost 3 times higher than that of sample A18. This excellent result can also be attributed to the formation of reinforcing cross-linked CNFs networks by coordination chemistry with divalent cations of Ca^2+^ inside the cross-linked alginate polymer structure. For this reason, the alginate composite synthesised with lower amounts of calcium chloride such as A3-CNFs showed lower reinforcement with respect to A3 because there was a smaller number of divalent cations to connect alginate chains and carbon nanofibers.

The reinforcement of calcium alginate achieved with the use of CNFs in this study is lower than that obtained in a previous study utilising the same amount of GO and calcium chloride [21]. This fact occurs because GO possess more oxygen functional groups than CNFs, and thus more cross-linking points. In that study, the enhancement of water diffusion achieved with the addition of 0.1% *w/w* of GO was also higher than that obtained here with 0.1% *w/w* of CNFs due to the presence of more carbon nanochannels in the dry state produced by the higher cross-linking between GO nanosheets.

Nevertheless, CNFs have much lower cost than GO (e.g., approximately 21 times more economical than GO when obtained from Sigma-Aldrich) and have attracted great interest in the field of regeneration medicine due to their excellent mechanical, magnetic and electrical properties [34], which renders these advanced composite materials very promising for certain biomedical and industrial applications in need of low-cost hydrogels with these required physical properties.

## 4. Conclusions

The effect of cross-linker content on water diffusion and compression performance was studied in carbon nanofibers/calcium alginate composite hydrogels synthesised with a very low amount of nanomaterial (0.1% *w/w*). The results of this study showed that a minuscule amount of CNFs can significantly enhance liquid water diffusion and compression in calcium alginate hydrogels due to the formation of connected CNFs networks by coordination chemistry inside the cross-linked alginate polymer matrix. This enhancement of properties increases with increasing cross-linker content due to the formation of cross-linked carbon nanochannels able to improve water diffusion and mechanical compression at the same time. These achievements demonstrate the potential utilisation of these new alginate composite hydrogels in many industrial applications currently in need of low-cost advanced hydrogels with enhanced compression and water diffusion properties.
